# lncRNAs as Biomarkers of Hepatocellular Carcinoma Risk and Liver Damage in Advanced Chronic Hepatitis C

**DOI:** 10.3390/cimb47050348

**Published:** 2025-05-10

**Authors:** Driéle B. dos Santos, Geysson J. Fernandez, Letícia T. Silva, Giovanni F. Silva, Estela O. Lima, Aline F. Galvani, Guilherme L. Pereira, Adriana C. Ferrasi

**Affiliations:** 1 Department of Internal Medicine, Medical School, Sao Paulo State University (UNESP), Botucatu 18618-687, SP, Brazil; driele.bretones@unesp.br (D.B.d.S.); leticia.toloto@unesp.br (L.T.S.); giovanni.f.silva@unesp.br (G.F.S.); estela.lima@unesp.br (E.O.L.); aline.galvani@unesp.br (A.F.G.); 2 Grupo Biología y Control de Enfermedades Infecciosas (BCEI), Universidad de Antioquia (UdeA), Medellín 050010, Colombia; geysson.fernandez@udea.edu.co; 3 Experimental Research Unity (UNIPEX), Faculty of Medicine, São Paulo State University (UNESP), Botucatu 18618-687, SP, Brazil; 4 Department of Animal Breeding and Nutrition, School of Veterinary e Animal Science (FMVZ), São Paulo State University (Unesp), Botucatu 18618-681, SP, Brazil; guilherme.luis@unesp.br

**Keywords:** hepatocellular carcinoma, chronic hepatitis C, lncRNA, biomarker

## Abstract

**Background/Objectives:** LncRNAs have emerged as promising biomarkers due to their role in gene regulation of carcinogenesis and presence in biological fluids. Liquid biopsies offer a less invasive alternative to tissue biopsies, improving early cancer diagnosis and surveillance. Hepatocellular carcinoma (HCC) is among the most lethal and prevalent cancers. Late diagnoses contribute to poor prognosis, particularly in chronic hepatitis C (CHC) patients, which is a major risk factor for HCC. Tissue biopsies for HCC diagnosis pose risks, including tumor dissemination, highlighting the urgent need for noninvasive biomarkers. Several lncRNAs are deregulated in HCC and may be potential markers for assessing HCC risk in CHC. This study evaluated seven lncRNAs as plasma biomarkers for HCC risk in CHC. **Methods:** lncRNA expression was analyzed by RT-qPCR in three groups: CHC patients who developed HCC within a 5-year follow-up (HCCpos), CHC patients who did not develop HCC within a 5-year follow-up (HCCneg), and healthy blood donors (CG). **Results:** This study found that plasma lncRNAs HULC and RP11-731F5.2 are potential biomarkers for HCC risk, while RP11-731F5.2 and KCNQ1OT1 may serve as noninvasive biomarkers for liver damage due to HCV infection. **Conclusions:** These findings highlight the potential of lncRNAs in enhancing early diagnosis and monitoring of HCC in CHC patients.

## 1. Introduction

Long noncoding RNAs (lncRNAs) are transcripts with at least 200 nucleotides that do not encode proteins [1,2,3]. These molecules regulate gene expression, interacting with other RNAs, DNA, and proteins of cellular cycle important pathways. Deregulation of lncRNA expression plays fundamental roles in tumor development and progression [4,5,6,7]. Consequently, lncRNAs are being studied as prognostic markers [8,9] and potential therapeutic targets in cancer [10,11]. Most of these studies are based on tissue biopsies; however, lncRNAs have also been found as circulating molecules in body fluids (e.g., plasma, serum, urine, saliva) [12]. In an oncological setting, tumor cells release lncRNAs either freely or within membrane microvesicles called exosomes [8,13,14,15], and since blood circulates throughout the body, it can serve as an important source of these molecules [16]. These lncRNAs can be used as tumor biomarkers, prognostic biomarkers, and for monitoring post-therapy progress.

Tissue biopsies are invasive, and their results can be influenced by sampling variability, inter-observer subjectivity, and tumor heterogeneity [17,18]. However, when a biomarker is detected in biofluids such as blood, the diagnostic approach can be minimally invasive, less heterogeneous, and, in some cases, early. This makes liquid biopsy studies particularly relevant for hepatocellular carcinomas (HCCs).

HCC is challenging to biopsy due to the risks involved, which, although rare, can be lethal; additionally, there is a possibility of tumor dissemination along the path of the percutaneous needle [19,20]. Thus, the primary diagnostic approach relies on imaging tests, such as ultrasound, and monitoring high-risk individuals through ultrasound and alpha-fetoprotein (AFP) serological levels [21]. However, even imaging tests have their limitations in accuracy and sensitivity, while common serum markers have low diagnostic performance, particularly during the early stages [22,23].

Chronic hepatitis B and C, chronic ethanol abuse, non-alcoholic fatty liver disease, and aflatoxin B1 exposure are the main factors involved in liver carcinogenesis [24]. Cirrhosis, regardless of etiology, is an independent risk factor for the development of HCC, especially in patients with chronic hepatitis C (CHC) [25,26]. Based on the natural history of CHC, there are estimates that 10% to 20% of patients will develop liver cirrhosis and 1% to 5% will develop HCC within 20 to 30 years [27]. Once liver cirrhosis is established, HCC develops at an annual rate of 5% to 7% [28]. Early diagnosis and treatment can prevent liver cirrhosis and HCC, especially with the application of screening and advanced treatment of CHC with direct-acting antiviral (DAA) therapy. However, the diagnosis of hepatitis and cancer is often late for a large proportion of the population, and the disease continues to represent a serious public health problem. According to World Health Organization (WHO) estimates, there were about 50 million people living with chronic hepatitis C in 2022 worldwide, highlighting its significant burden [29].

Due to the lack of specific markers and absence of clinical symptoms, most patients are already in advanced stages of HCC when they are diagnosed, thereby negatively impacting the prognosis [30]. The overall 5-year survival rate for all stages of HCC is only 15% [31]; yet, if diagnosed early, it can reach 70% [30,32,33]. Therefore, noninvasive biomarkers for the early diagnosis of HCC are urgently needed. Several lncRNAs have been found to be deregulated in HCC [34,35], and in a next-generation sequencing (NGS) study conducted by our group, some deregulated lncRNAs were detected in liver tissue from patients with advanced CHC and HCC compared with healthy liver tissues [36]. Since certain HCC-related lncRNAs are present in body fluids [37,38], the seven top-ranked deregulated lncRNAs observed in our previous study [36] were evaluated as potential plasma biomarkers for HCC risk in patients with advanced CHC, using real-time quantitative PCR (RT-qPCR).

## 2. Materials and Methods

An overview of the study workflow is presented in the Supplementary Materials Figure S1.

Patients: This study was approved by the São Paulo State University Research Ethics Committee (CAAE 30691220.7.0000.5411, approved on 20 May 2020) and was conducted in accordance with the relevant guidelines/regulations for research involving human participants (*n* = 63) and in accordance with the Declaration of Helsinki. Informed consent was obtained from all subjects involved in the study.

Plasma samples from volunteer participants were obtained from peripheral blood centrifuged at 704× *g* (RCF) for 10 min. All samples were stored at −70 °C until use. A total of 41 volunteer participants with advanced CHC, who underwent clinical follow-ups for a minimum of five years after plasma collection, were selected for the study. The volunteer inclusion criteria were as follows: patients > 18 years old, unrelated, diagnosed by detection of HCV-RNA, treatment-naïve HCV before sample collection, and with advanced fibrosis (METAVIR F3/F4). Fibrosis was classified based on the METAVIR score by percutaneous biopsy [39]. Diagnosis of HCC was based on clinical symptoms, imaging studies (ultrasound, CT, and MRI), serum AFP levels, and histopathological examinations. Volunteers with other liver diseases or HBV or HIV coinfection were excluded. This study also included 22 healthy volunteer blood bank donors with no history of HCC or HCV infection and with healthy physical examinations (healthy control group [CG]). The participants were categorized into three biological groups (BioGroup): (i) HCCpos and (ii) HCCneg, according to the development or non-development of HCC, respectively, within a 5-year follow-up, as well as (iii) CG (healthy control group). The three study groups were balanced for age and sex. After sample collection, all HCV patients received DAA therapy and remained on sustained virologic response until the end of follow-up. The demographic and clinical characteristics of the study participants are summarized in Table 1.

RT-qPCR: Total RNA was isolated from a 500 μL plasma sample using Plasma/Serum Circulating and Exosomal RNA Purification Mini Kit (Norgen Biotek Corp., Thorold, ON, Canada) according to the manufacturer’s protocol. RNA samples were treated with Turbo DNase (Life Technologies Corp. Carlsbad, CA, USA) to remove genomic DNA contamination. RNA was reverse transcribed to cDNA by High-Capacity cDNA Reverse Transcription Kit (Thermo Fisher Scientific, Waltham, MA, USA), and RT-qPCR was performed using the Power SYBR Green PCR Master Mix (Thermo Fisher Scientific) with StepOne Plus^TM^ System (Applied Biosystems, Foster City, CA, USA), according to the manufacturer’s protocol. Briefly, RT-qPCR conditions were as follows: initial denaturation at 95 °C for 2 min, followed by 40 cycles of 95 °C for 15 sec and 62 °C for 1 min. Primer sequences are listed in Table 2. The lncRNA expression level was calculated using the 2^−ΔΔCt^ method [40] and β-actin was selected as an internal reference. The specificity of assays was confirmed by dissociation melting curve and polyacrylamide gel electrophoresis. Samples were analyzed in triplicate, with no-template controls included.

Statistical Analysis: Assumptions for the use of parametric statistics were tested (Shapiro–Wilk test and Levene test). For the data complied with the assumptions, ANOVA or Student’s *t*-tests were performed. For the data that did not comply with the assumptions, the nonparametric Kruskal–Wallis test or Mann–Whitney test was used. Receiver operating characteristic (ROC) curves and Pearson’s correlation test were used. All statistical analyses were conducted using GraphPad v. 9.5.1 (GraphPad Software Inc., San Diego, CA, USA). Statistical significance was set at *p* < 0.05. ROC curves were subjected to combinatorial analysis using CombiROC online tool [41] (http://combiroc.eu/, accessed on 28 March 2024).

## 3. Results

From a previous next-generation sequencing (NGS) study [36] of liver tissue from patients with advanced CHC, HCC, and healthy liver tissue, the seven top-ranked deregulated lncRNAs were selected to be evaluated as potential plasma biomarkers of HCC risk in patients with advanced CHC, using real-time quantitative PCR (RT-qPCR). The fold changes from the previous study are available in Ferrasi et al. [36].

**Figure 1:**
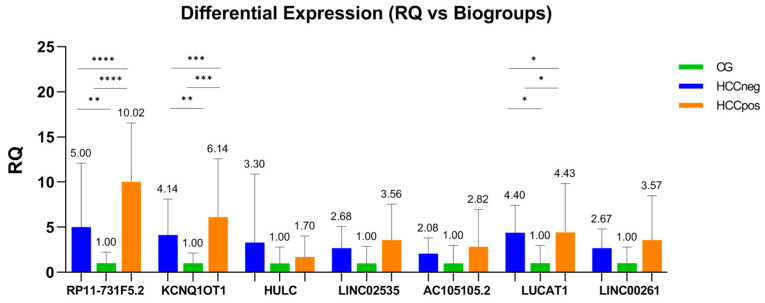
Differential expression (RQ) of lncRNAs when the HCCneg and HCCpos groups are compared to healthy donors (CG). RQ calculated by the comparative CT method (2^−∆∆CT^). Differential expression (RQ of HCCneg and HCCpos, respectively) of RP11-731F5.2 (5.00 and 10.02), KCNQ1OT1 (4.14 and 6.14); HULC (3.30 and 1.70), LINC02535 (2.68 and 3.56), AC105105.2 (2.08 and 2.82), LUCAT1 (4.40 and 4.43), and LINC00261 (2.67 and 3.57). The ANOVA or Kruskal–Wallis test, as well as the Student’s *t*-tests or Mann–Whitney test, were used following results from the Shapiro–Wilk test and Levene test. * *p* < 0.05; ** *p* < 0.01; *** *p* < 0.001; **** *p* < 0.0001.

**Figure 2:**
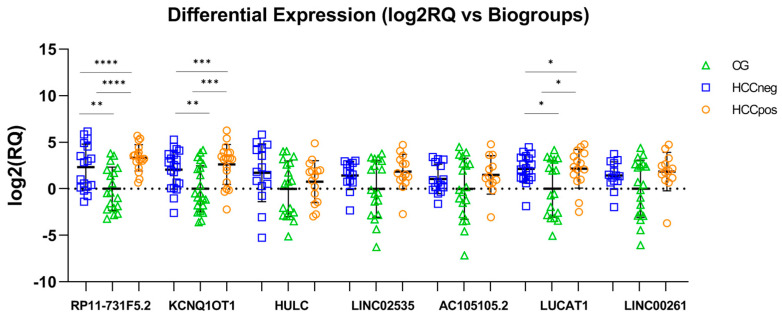
Scatter plots show the log2(RQ) values of lncRNAs when HCCneg and HCCpos are compared to healthy donors (CG). The mean log2(RQ) values and standard deviation are shown in each scatter plot. All lncRNAs were upregulated in both HCCneg and HCCpos groups compared to the CG group, particularly RP11-731F5.2, KCNQ1OT1, and LUCAT1. The ANOVA or Kruskal–Wallis test, as well as the Student’s *t*-tests or Mann–Whitney test, were used following results from the Shapiro–Wilk test and Levene test. * *p* < 0.05; ** *p* < 0.01; *** *p* < 0.001; **** *p* < 0.0001.

The present study compared three groups: the HCC-negative (HCCneg), HCC-positive (HCCpos), and healthy control groups (CG). The results showed that all selected lncRNAs were upregulated in both the HCCneg and HCCpos groups compared with the CG group (Figure 1 and Figure 2).

Also, CHCneg and CHCpos were compared to each other, and in HCCpos cases, the expression of RP11-731F5.2 was observed to be twice that in CHCneg cases (RQ = 2.004). Similar trends were noted for KCNQ1OT1 (RQ = 1.484), AC105105.2 (RQ = 1.354), LINC02535 (RQ = 1.330), and LINC00261 (RQ = 1.340). lncRNA HULC was downregulated in HCCpos cases compared to HCCneg (RQ = 0.520). There was no significant variation in LUCAT1 expression between the two groups (RQ = 1.010). These data are presented graphically as Supplementary files (Figure S2A,B).

No correlation was observed between the expression of lncRNAs and age, gender, body mass index (BMI), degree of fibrosis, or viral subtype (HCV) (Pearson’s correlation).

**Figure 3:**
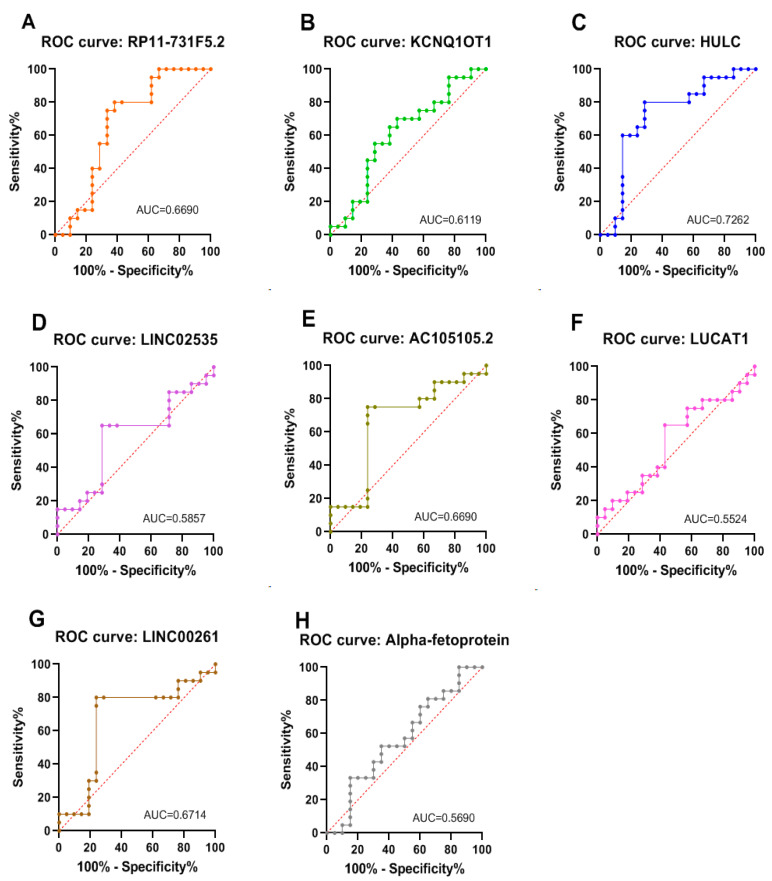
ROC Curve Analysis for lncRNAs in Plasma. CHCneg versus CHCpos groups; IC = 95%; AUC: Area Under Curve; the red dashed line represents the reference line (nullity). This analysis and graphs were performed in GraphPad Prism version 9.5.1. (**A**) RP11-731F5.2 (AUC = 0.6690, *p* = 0.0541), sensitivity (80%) and specificity (62%); (**B**) KCNQ1OT1 (AUC = 0.612, *p* = 0.2203), sensitivity (70%) and specificity (57%); (**C**) HULC (AUC = 0.726, *p* = 0.0132), sensitivity (73%) and specificity (71.5%); (**D**) LINC02535 (AUC = 0.586, *p* = 0.3478), sensitivity (65%) and specificity (71.5%); (**E**) AC105105.2 (AUC = 0.669, *p* = 0.0541), sensitivity (75%) and specificity (76%); (**F**) LUCAT1 (AUC = 0.552, *p* = 0.5661), sensitivity (65%) and specificity (57%); (**G**) LINC00261 (AUC = 0.671, *p* = 0.0475), sensitivity (80%) and specificity (76%); (**H**) alpha-fetoprotein (AUC = 0.569, *p* = 0.4494), sensitivity (85%) and specificity (33%).

ROC curve analysis was performed to differentiate the HCCpos group from the HCCneg group. In this analysis, an area under the curve (AUC) closer to 1 indicates a more significant marker for distinguishing between the two groups [42]. Figure 3 shows the ROC curves constructed for each lncRNA and the plasmatic AFP. HULC was the best biomarker of HCC risk (AUC = 0.726), followed by LINC00261 (AUC = 0.671), RP11731F5.2 (AUC = 0.669), AC105105.2 (AUC = 0.669), KCNQ1OT1 (AUC = 0.612), LINC02535 (AUC = 0.586), and LUCAT1 (AUC = 0.552). AFP was the least accurate marker (AUC = 0.569) in the series analyzed in this study.

In addition, using the CombiROC [41] online tool (http://combiroc.eu/, accessed on 28 March 2024), we combined the biomarkers (two by two or three by three) and repeated the ROC analysis to improve accuracy. The results of the head arrangements are summarized in Table S1 (Supplementary file). Some combinations of markers resulted in a higher AUC than that obtained using only one lncRNA; for example, the lncRNA combination of RP11731F5.2/HULC (AUC = 0.779) had improved sensitivity and specificity (99% and 62%, respectively).

Another ROC curve analysis was performed to distinguish HCV-positive samples from control samples (CG), evaluating the resulting lncRNAs as biomarkers of liver damage due to HCV infection. In this context, the best biomarkers were RP11731F5.2 (AUC = 0.907; *p* < 0.0001), followed by KCNQ1OT1 (AUC = 0.815; *p* = 0.0004), LUCAT1 (AUC = 0.745, *p* = 0.0059), LINC02535 (AUC = 0.685; *p* = 0.0385), LINC00261 (AUC = 0.663; *p* = 0.0004), AC105105.2 (AUC = 0.6587; *p* = 0.0059), and HULC (AUC = 0.5891; *p* = 0.3181). The ROC curves are available as Supplementary files Figure S3A,B.

A linear correlation between the levels of the studied biomarkers was verified using Pearson correlation. The samples were distributed among the cases grouped by HCC status (Table 3). 

## 4. Discussion

Recurrence-free survival and the prognosis of HCC are critically dependent on the stage of the disease at diagnosis [43,44]. Its high mortality rate is mainly related to the absence of precise symptoms in the early stages [45]. Thus, the identification of biomarkers is essential for early diagnosis and treatment.

Several lncRNAs show differential expressions between cancerous and healthy tissues, highlighting their role in carcinogenesis and tumor progression. A growing number of experiments have demonstrated a close connection between lncRNAs and HCC [46,47].

Although most studies on lncRNAs have focused on tissue biopsy; recently, these transcripts have been differentially detected in body fluids, positioning them as promising candidates for liquid biopsy biomarkers [48]. This scenario has driven several studies on lncRNAs as plasma and serological prognostic markers in hepatocellular carcinoma [49]. For example, lncRNA-WRAP53 in serum was shown to be an independent prognostic marker for predicting a high recurrence rate in patients with HCC [50]. Furthermore, serum LINC0052 expression was found to be an independent survival factor in patients with HCV-related HCC [51]. Recently, Samir et al. [52] employed machine learning to evaluate the plasma levels of four lncRNAs (LINC00152, LINC00853, UCA1, and GAS5) as biomarkers for HCC and prognosis. Higher LINC00152 expression levels and lower GAS5 expression levels were correlated with increased risk of mortality. Furthermore, the integration of lncRNA biomarkers with conventional laboratory data (e.g., ALT, AST, and AFP) demonstrated significant potential for a precise and cost-effective diagnostic tool for HCC.

In this context, the present study analyzed the plasma levels of seven lncRNAs that were previously reported by our group to be differentially expressed in liver tissue samples from patients with advanced CHC and HCC [35], investigating the potential association with increased risk of developing HCC and liver damage due to HCV infection.

The plasma level of all lncRNAs investigated was higher in patients with CHC (both HCCneg and HCCpos) when compared to healthy controls (CG) (Figure 1 and Figure 2). A previous study associated lncRNAs with liver injury characterized by hepatocyte damage, marked inflammatory responses, and fibrosis [53]. RP11-731F5.2 is located on chromosome 14, and its expression in tumor diseases needs further elucidation. Because it was differentially expressed in liver tissue from CHC and HCC patients in our previous study [35], we evaluated RP11-731F5.2 in plasma samples from healthy donors and patients with advanced CHC. This transcript was 5- and 10-fold upregulated (HCCneg and HCCpos, respectively) in CHC patients compared to healthy controls. These results highlight its potential as a marker of liver injury. According to our knowledge, RP11-731F5.2 (Ensembl gene ENSG00000253364 and alias COPDA1) has been investigated in only three published studies. Recently, Xu et al. [54] reported that positive regulation of COPDA1 increases intracellular reactive oxygen species (ROS) levels, and influences proliferation, migration, and invasion in two melanoma cell lines and clinical tissues. Jing et al. [55] concluded that the transcript is an accomplished serum biomarker for diagnosis and prognosis in gastric tumors, in addition to having verified its stability in serum, even after being placed at room temperature for 24 h and after repeated freezing–thawing seven times. Also, RP11-731F5.2 was found to be involved in the progression of chronic obstructive pulmonary disease (COPD) [56]. This study demonstrated that RP11-731F5.2 (COPDA1) promoted the proliferation of human bronchial smooth muscle cells by upregulating the expression of MS4A1 (membrane-spanning 4-domains family, subfamily A) and the levels of cyclin D1 protein and phosphorylated RB (pRB).

The cyclin D1/pRb pathway plays an important role in cell cycle progression; cyclin D1 can bind and activate CDK4 (Cyclin-dependent kinase 4), which then phosphorylates pRB, subsequently activating E2F proteins and the expression of target genes that are necessary for promoting the G1/S phase transition [57,58]. This pathway is considered relevant in liver carcinogenesis and as a therapeutic target [59,60,61]. Future studies may reveal that the deregulation of RP11-731F5.2 is a key component in this network of interactions in HCCs. MS4A1 encodes the CD20 protein, which is part of the Ca^2+^ channels in normal and malignant B cells, regulating the activity and proliferation of these cells [62]. Interestingly, a previous study that aimed to evaluate the role of tumor-infiltrating B cells (B-TILs) in the clinical response to HCC treatment found that this cancer was highly CD20+ B cells-enriched and that CD20 expression is elevated in tumor tissues compared to peritumoral ones [63]. Furthermore, this study found an association between high infiltration of CD20+ B cells and impaired antitumor activity, characterized by CD8+ T cells and NK cells with reduced capacity to express granzyme B and IFN-γ [63]. Taken together, the data highlight the importance of the elevated expression of RP11-731F5.2 (COPDA1) in HCCpos patients (RQ = 10.023) in our study. However, this hypothesis is based on studies conducted in other cases, and additional research is required to confirm its validity.

Besides RP11-731F5.2, KCNQ1OT1 and HULC were identified as prominent lncRNAs. KCNQ1OT1 plays a vital role in the development and progression of several types of cancer, including HCC [64]. KCNQ1OT1 is upregulated in HCC tissues and cell lines [65]. Emerging evidence shows that KCNQ1OT1 functions as a competing endogenous RNA sponge for several miRNAs in HCC, such as miR-148a-3p [65], miR-149 [66], miR-29a-3p [67], miR-7-5p [68], and miR-504 [69]. All such studies concluded that overexpression of KCNQ1OT1 was associated with HCC cell growth and may be potential new therapeutic targets for patients with high intra-tumor transcript levels. According to the available literature, the current study is the first to analyze the plasma expression of KCNQ1OT1 in CHC. HULC (highly upregulated in liver cancer), encoded on chromosome 6p24.3, was first identified in HCC samples as a novel lncRNA markedly upregulated compared to non-tumor liver tissue samples [70]. Since then, it has been investigated for its role in liver carcinogenesis and as a serological and plasma marker for early diagnosis of HCC [50,51,71]. The findings revealed upregulation in CHC plasma compared to healthy controls (Figure 1 and Figure 2), corroborating other studies in plasma or serum from patients with HCV [72,73] and HBV-related HCC [71]. Furthermore, Gaber et al. also found that HULC was highly upregulated in both the HCV and HCC groups, with higher levels in the HCC group compared to the HCV group [74]. This lncRNA has been implicated in several other cancers [75,76] and can regulate several miRNAs in HCC cells, including miR-372 [77], miR-186 [78], miR-150-5p [79], and miR-3200-5p [80], in addition to downregulating the tumor suppressor p18 [81].

The HCCneg and HCCpos groups were compared to determine if plasma levels of these lncRNAs could distinguish patients with advanced hepatitis C at higher risk of progression to HCC. HULC was the best marker for this purpose (AUC = 0.726; sensitivity [73%] and specificity [71.5%]), followed by lncRNA RP11-731F5.2 (AUC = 0.669; sensitivity [80%] and specificity [62%]) (Figure 3). These results highlight HULC as a relevant marker for further studies in translational medicine. Surprisingly, HULC was downregulated in plasma samples from the HCCpos group compared to HCCneg (RQ = 0.520). A relatively similar result was observed in our previous NGS study [36], where we found that HULC was downregulated in HCC liver tissue samples when compared to normal tissue. These results contrast with most published studies, which report the upregulation of this lncRNA in HCC [70,77,82]. Although the mechanisms underlying HULC upregulation in many cancer types require elucidation, many studies have analyzed hepatitis B-related HCC. Du et al. demonstrated that HBV X protein, an oncogenic viral protein involved in HBV pathogenicity, upregulates HULC and thus promotes hepatoma cell proliferation in vitro and in vivo [82]. Studies of HULC in HCV-related carcinoma are scarce; however, the discordance of our results (in two independent studies and with different technical approaches) may indicate that HULC deregulation in HCV-HCC may occur through pathways other than HBV-HCC, prompting further investigation.

Other studies have also evaluated HULC as a biomarker of HCC in serum or plasma [71,72,73]. However, this study is the first to assess it as a biomarker for HCC risk in the plasma of patients with advanced CHC followed for at least 5 years.

The potential for combinations of lncRNAs (two by two or three by three) to improve accuracy in distinguishing between risk groups was also evaluated (Table S1; Supplementary file); some combinations achieved a higher AUC than that obtained with the use of HULC or RP11-731F5.2 by themselves, for example, in the HULC/RP11-731F5.2 combination (AUC = 0.779; sensitivity [99%] and specificity [62%]). However, this does not justify the increased financial and technical costs associated with adding one or two more markers, as two markers alone have already demonstrated good performance (Figure 3). The combinations of lncRNAs with AFP levels were also evaluated; however, all combinations failed to indicate a risk group for HCC. Plasma AFP is the most widely used biomarker for HCC, although AFP is limited by its low sensitivity and specificity, especially in early-stage HCC [82,83,84]. It is acknowledged that AFP plasma level is not considered a predictive risk marker for HCC. However, this initial hypothesis remains relevant, given the widespread use of AFP as a marker for monitoring hepatic lesions, including HCC. It is noteworthy that this marker alone was the least accurate marker (AUC = 0.569) in the series analyzed in this study.

A risk biomarker indicates an individual’s increased likelihood of developing a disease or condition before clinical manifestation [85]. They play a crucial role in clinical practice, guiding preventive strategies and identifying individuals who require more intensive disease surveillance. An ideal biomarker should be quantifiable, sensitive, and specific, with results generated quickly through assays adaptable to clinical practice, using easily accessible specimens [86]. In light of this and the impact of early diagnosis of HCC on survival, the findings suggest that plasma levels of the HULC and RP11-731F5.2 hold promise as risk biomarkers for HCC.

The regulation of lncRNAs in HCC remains poorly understood. To expand knowledge on this subject, the relationship among plasma levels of the studied lncRNAs was investigated (Table 3). A striking positive correlation between AC105105.2 and LINC02535 was found in both HCCpos (R = 0.89; *p* < 0.001) and HCCneg (R = 0.91; *p* < 0.001) groups. AC105105.2 (alias MIR122HG) is a precursor of microRNA-122, a class of lncRNAs called miRNA-host gene-derived lncRNAs (lnc-MIRHGs). Information about this transcript is scarce, but an overexpression and silencing study in Balb/c mice confirmed its protective role in acute injury by promoting hepatocyte proliferation in vivo and in vitro. Furthermore, MIR122HG promoted the transcription of CXC chemokines and thereby activated signaling pathways that stimulate the proliferation of new healthy liver cells [87]. The CXC chemokine family plays a significant role in liver injury and regeneration [88].

Apart from this current investigation, the only other report of MIR122HG in humans was published by Dhir et al. [89], whose aim was to report that most lnc-MIRHG do not use the canonical cleavage and polyadenylation pathway; instead, they employed microprocessor cleavage to terminate transcription. LINC02535 was found to be upregulated in poorly differentiated gastric cancer [90] and cervical cancer [91], where it was shown to directly interact with poly-binding protein 2 (PCBP2) in the cytoplasm, regulating cell proliferation, DNA damage repair, and tumor progression. Furthermore, LINC02535 is upregulated in lung cancer and acts as a sponge by inhibiting miR-30a-5p [92], which, interestingly, is downregulated in serum and liver tissue of HCC patients [93]. A strong correlation was also observed between LINC02535 and LINC00261 in both HCCpos (R = 0.91) and HCCneg (R = 0.93) groups (*p* < 0.001). Additionally, AC105105.2 and LINC00261were strongly correlated (R = 0.88; *p* < 0.001) across both groups.

LINC00261 is abnormally expressed in a variety of tumors such as gastric, colorectal, lung, breast, laryngeal, prostate, endometrial, esophageal, prostate, cholangiocarcinoma, and hepatocellular carcinoma [94]. This transcript acts as a tumor suppressor by regulating cell proliferation, apoptosis, chemoresistance, and tumorigenesis, with its upregulation indicating favorable prognoses [94]. LINC00261 binds to miR-552-5p [95], miR-23a-3p [96], and miR-222-3p [97] to promote the expression of tumor-suppressor genes to inhibit cancer progression. Through bioinformatics analysis, Zhu et al. [98] showed that LINC00261 might interact with miRNA-23b-3p, miRNA-211-5p, miRNA-205-5p, miRNA-140-3p, and miRNA-125b-5p in melanoma. Thus, HULC [80], LINC02535 [92], LINC00261 [95,96,97,98], and KCNQ1OT1 [64,99] may act as competing endogenous RNAs (ceRNAs) in various cancers, including HCC. ceRNAs are transcripts that regulate gene expression and play a vital role in tumor development by inhibiting miRNA function through competitive binding to multiple miRNAs. In other words, ceRNAs can sponge miRNAs specifically and effectively [100,101]. Any RNA molecule can act as a ceRNA, as long as it has a binding affinity with miRNA [100,101], and can include mRNAs, pseudogenic RNAs [102], circRNAs [103], and lncRNAs [104]. LncRNAs play many fundamental roles in both diseases and healthy physiology, particularly in relation to cancer. Their functions in carcinogenesis are partially attributed to their role as ceRNAs, which have the potential as cancer biomarkers.

To date, there are no studies reporting associations between the three lncRNAs (LINC02535, AC105105.2, and LINC00261). However, the strong correlation in the plasma expression of these lncRNAs should encourage future studies to clarify their combined role in relevant molecular pathways involved in liver carcinogenesis.

An ideal biomarker satisfies the following properties: it should be either binary (i.e., present or absent) or quantifiable without subjective assessments; the result should be generated by an assay that is adaptable to routine clinical practice and has a timely turnaround (i.e., in a matter of days rather than weeks); the biomarker assay should be sensitive and specific; and most importantly, the biomarker should be detectable using easily accessible specimens.

The limitations of the present study include its small sample size and the fact that it was a single-center study. Future research efforts on this topic should focus on validation in sizeable and more diverse patient cohorts, which can increase the reliability and applicability of the candidate biomarkers. Furthermore, future studies are needed to confirm that these transcripts are exclusive markers for HCC risk and that they are not present in other liver diseases.

## 5. Conclusions

In conclusion, this study, to the best of our knowledge, is the first to focus on lncRNAs as potential biomarkers of hepatocellular carcinoma risk in chronic hepatitis C. The results revealed that plasmatic lncRNAs HULC and RP11-731F5.2 are potential biomarkers of HCC risk, and RP11-731F5.2 and KCNQ1OT1 are possible noninvasive biomarkers of liver damage due to HCV infection. Additional studies with a larger and more diverse cohort are needed to confirm these findings.

## Figures and Tables

**Table 1:** Demographic and clinical characteristics of all study participants.

Variables		BioGroups		
	Control Group *n* = 22	HCCneg *n* = 21	HCCpos *n* = 20	*p*-Value ^†^
**Age (years)**	57.2 ± 7.5	56 ± 9.0	58.9 ± 6.7	0.999
**Sex**				
Male	12 (54.5)	12 (57%)	15 (75%)	0.339
Female	10 (45.5)	9 (43%)	5 (25%)	
**BMI (Kg/m^2^)**	25.6 ± 7.4	28.2 ± 5.8	27.2 ± 4.6	
**HCV Genotype**				
1 *	-	17 (81%)	14 (70%)	0.484
Not 1 **	-	4 (19%)	6 (30%)	
**Fibrosis Grade ^#^**				
F3	-	3 (14%)	3 (15%)	1.000
F4	-	18 (86%)	17 (85%)	
**AFP (ng/mL)**				
Median	-	7.2 (2.7–74.2)	9.6 (1.3–591)	0.457
Mean	-	19 ± 19.8	66 ± 140.7	
**HCC Diagnosis** ^##^				
Median	-	-	24 (8–89)	
Mean	-	-	35.5 ± 25.9	

^#^ METAVIR Score; control group: healthy participants; HCCneg: patients with chronic hepatitis C (CHC) who did not develop hepatocellular carcinoma (HCC) within 5 years of follow-up; HCCpos: patients with CHC who developed HCC within 5 years of follow-up; BMI: body mass index; HCV: hepatitis C virus; * HCV 1: hepatitis C virus genotype 1; ** HCV not 1: hepatitis C virus other genotypes; AFP: alpha-fetoprotein. ^##^ HCC diagnosis: interval (months) from sample collection to HCC diagnosis; ^†^ Chi-square test or Fisher’s exact test.

**Table 2:** Primer sequences for RT-qPCR analysis.

Target	Ensembl ID	Primers (5′ to 3′)	Amplicon (bp)
RP11-731F5.2	ENSG00000253364	F-TTCAGTCTTTGCAGCGTGGAG	121
		R-CCTGTTTTGGCGCGGTA	
KCNQ1OT1	ENSG00000269821	F-TGCAGAAGACAGGACACTGG	125
		R-CTTTGGTGGGAAAGGACAGA	
HULC	ENST00000503668	F-ACTCTGAAGTAAAGGCCGGA	95
		R-GCCAGGAAACTTCTTGCTTGT	
LINC02535	ENST00000455071	F-AAGGAGCTCTGTTCTCCAGG	102
		R-GCCTCTATGTAGGGCGCTTT	
AC105105.2	ENSG00000267391	F-CCCGTGATGCTTCTTTTCTC	150
		R-CCATTGTCACACTCCACAGC	
LINC00261	ENSG00000259974	F-TCAGATTGCTCCTGGACACTT	91
		R-GGACCATTGCCTCTTGATTAG	
LUCAT1	ENSG00000248323	F-GCTCGGATTGCCTTAGACAG	114
		R-GGGTGAGCTTCTTGTGAGGA	
β-ACTIN	ENSG00000075624	F-AGAGCCTCGCCTTTGCCGATCC	103
		R-CACATGCCGGAGCCGTTGTCG	

F: Forward; R: Reverse; bp = base pair.

**Table 3:** Pearson’s correlation matrix of the lncRNA in plasma, distributed among the cases grouped by HCC status.

	RP11-731F5	KCNQ1OT1	HULC	LINC02535	AC105105.2	LUCAT1	LINC00261	AFP
**RP11-731F5**	-	**0.70**	0.18	**0.50**	**0.58**	0.41	**0.46**	−0.13
**KCNQ1OT1**	**0.48**	-	0.40	**0.57**	**0.66**	**0.59**	**0.57**	−0.18
**HULC**	0.37	**0.76**	-	0.32	0.36	0.32	0.26	−0.01
**LINC02535**	0.37	**0.66**	0.33	-	**0.91**	**0.64**	**0.93**	−0.36
**AC105105.2**	**0.46**	**0.65**	0.32	**0.89**	-	**0.53**	**0.88**	−0.40
**LUCAT1**	**0.63**	**0.59**	0.43	**0.75**	**0.76**	-	**0.62**	0.04
**LINC00261**	0.33	**0.60**	0.24	**0.91**	**0.88**	**0.80**	-	−0.38
**AFP**	−0.09	0.07	−0.002	0.34	0.39	0.23	0.28	-

Shaded values (r coefficient) show the results from the HCCpos study group and unshaded values are from the HCCneg study group; correlation strength: negligible (r ≤ 0.3), low (0.3 < r ≤ 0.5), moderate (0.5 < r ≤ 0.7), high (0.7 < r ≤ 0.9), and very high (0.9–1.0). r values bold were *p*-value < 0.05; AFP: alpha-fetoprotein.

## Data Availability

The datasets generated during the current study are available from the corresponding author upon request.

## Supplementary Materials

The following supporting information can be downloaded at: https://www.mdpi.com/article/10.3390/cimb47050348/s1.

## Abbreviations

The following abbreviations are used in this manuscript:

ALT	Alanine Aminotransferase
AST	Aspartate Aminotransferase
AFP	alpha-fetoprotein
AUC	area under curve
B-TILs	tumor-infiltrating B cells
cDNA	complementary DNA
ceRNA	competing endogenous RNAs
CHC	chronic hepatitis C
circRNA	circular RNA
CT	computed tomography
DAA	direct-acting antiviral
HCC	hepatocellular carcinoma
HCV	hepatitis C virus
miRNA	microRNA
MRI	magnetic resonance imaging
RCF	relative centrifugal force
ROC	receiver operating characteristic
RQ	relative quantification
RT-qPCR	real-time quantitative reverse transcription PCR
