# Supplementary material for: lncRNAs as Biomarkers of Hepatocellular Carcinoma Risk and Liver Damage in Advanced Chronic Hepatitis C
Source: Curr Issues Mol Biol. 2025 May 10;47(5):348. doi: 10.3390/cimb47050348 (PMC12110020; doi:10.3390/cimb47050348)
Supplement: Supplementary file 1 [file cimb-47-00348-s001.zip › cimb-3569240-supplementary.pdf]

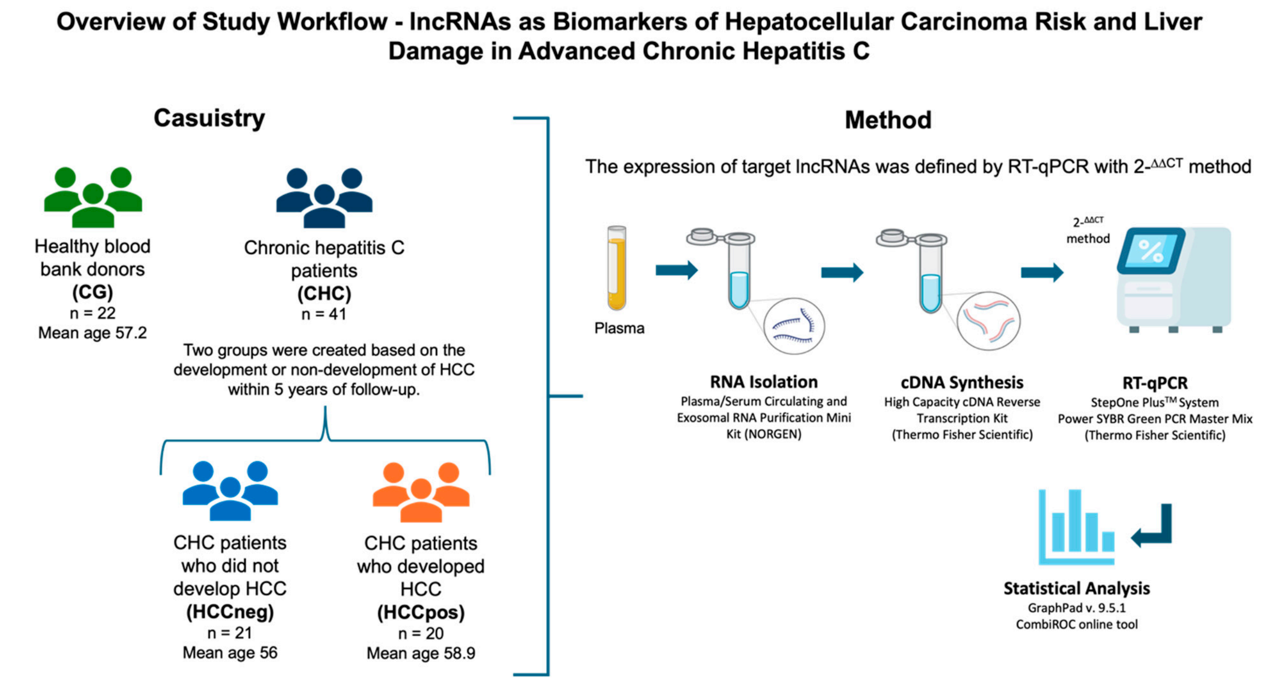

Figure S1. Overview of Study Workflow - lncRNAs as Biomarkers of Hepatocellular Carcinoma Risk and Liver Damage in Advanced Chronic Hepatitis C

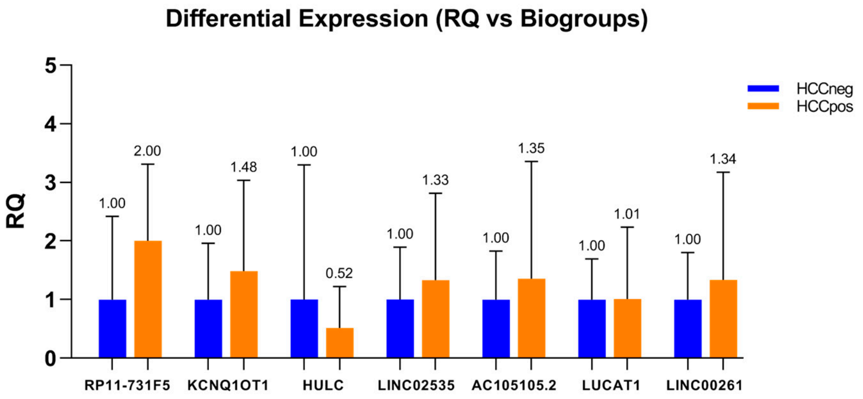

Figure S2A. Differential expression (RQ) of lncRNAs when HCCneg and HCCpos biogroups are compared. RQ calculated by the comparative CT method ($2^{-\Delta\Delta CT}$). Differential expression (RQ) of RP11-731F5.2 (2.00), KCNQ1OT1 (1.48); HULC (0.52), LINC02535 (1.33), AC105105.2 (1.35), LUCAT1 (1.01) and LINC00261 (1.34).

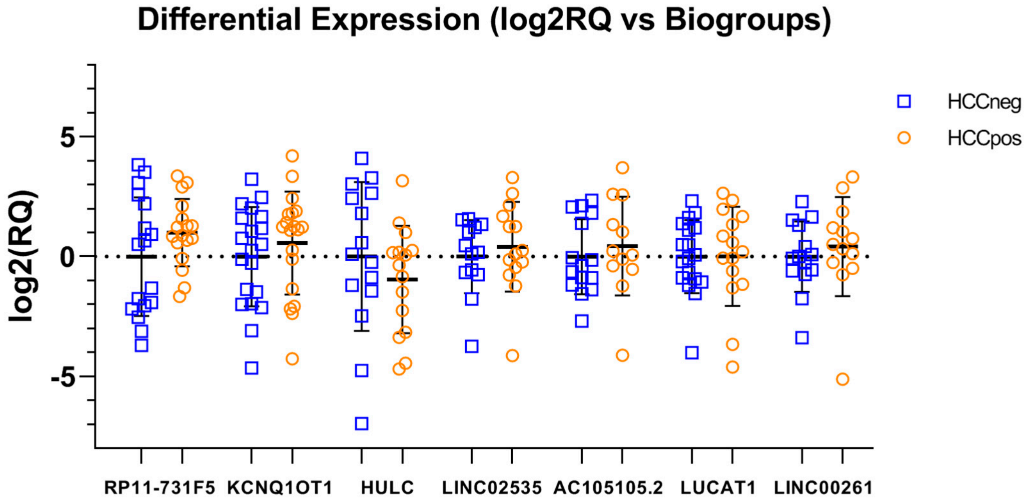

Figure S2B. Scatter plots show the log2(RQ) values of lncRNAs when HCCneg and HCCpos biogroups are compared. The mean log2(RQ) values and standard deviation is shown in each scatter plot. RP11-731F5.2 expression was twofold higher in HCCpos compared to HCCneg. In contrast, HULC was downregulated in the HCCpos group. The other lncRNAs exhibited slight upregulation in HCCpos, whereas LUCAT1 showed no difference in expression between the two groups.

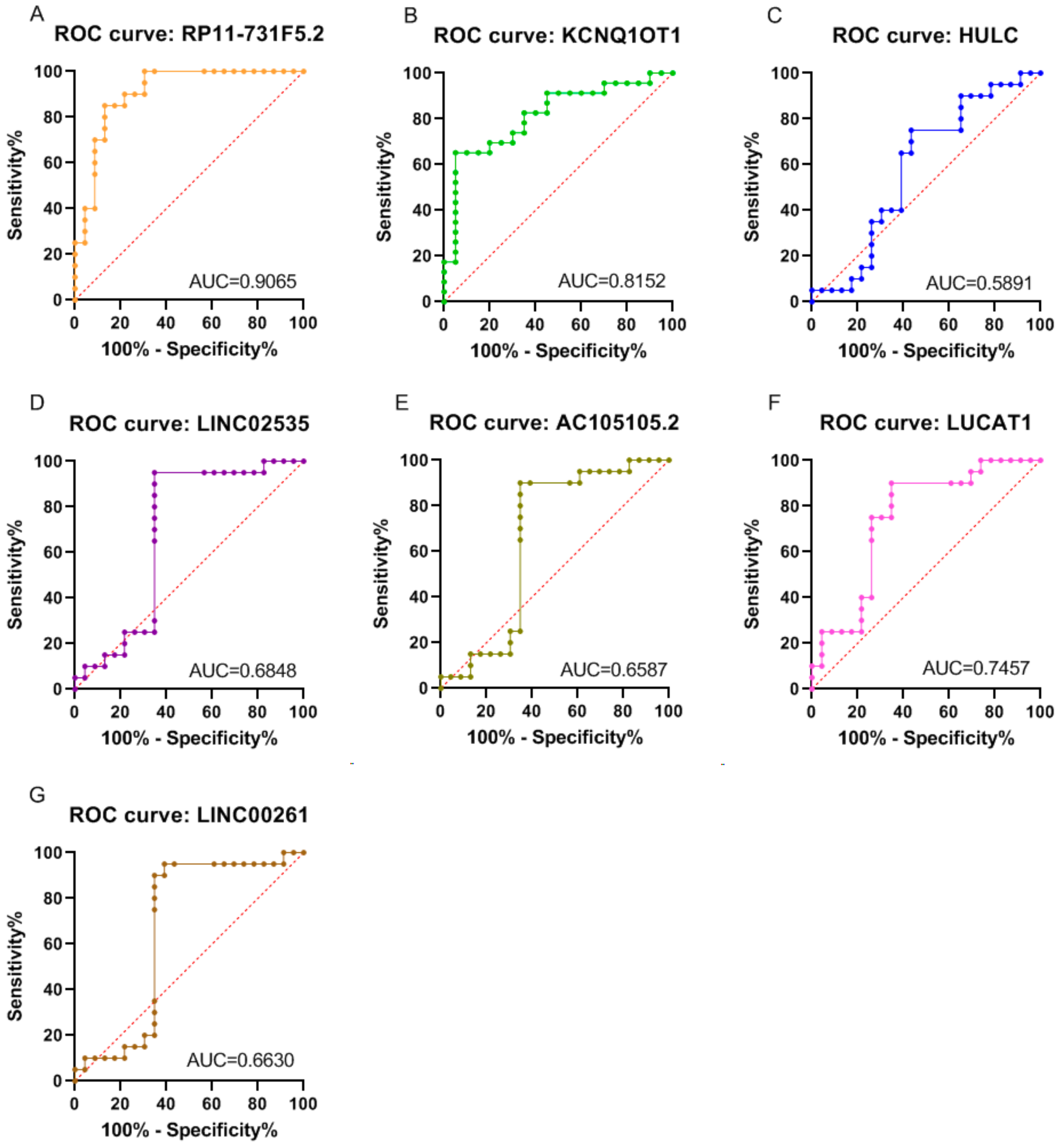

Figure S3A. ROC Curve Analysis for lncRNAs in Plasma. CHCpos *versus* DS groups; IC = 95%; AUC: Area Under Curve; The red dashed line represents the reference line (nullity). This analysis and graphs were performed in GraphPad Prism version 9.5.1. A) RP11-731F5.2 (AUC = 0.9065, p < 0.0001), sensitivity (85%) and specificity (87%); B) KCNQ1OT1 (AUC = 0.8152, p = 0.0004), sensitivity (95%) and specificity (65%); C) HULC (AUC = 0.5891, p = 0.3181), sensitivity (75%) and specificity (57%); D) LINC02535 (AUC = 0.6848, p = 0.0385), sensitivity (90%) and specificity (57%); E) AC105105.2 (AUC = 0.6587, p = 0.0755), sensitivity (95%) and specificity (65%); F) LUCAT1 (AUC = 0.7457, p = 0.0059), sensitivity (95%) and specificity (65%); G) LINC00261 (AUC = 0.6630, p = 0.0678), sensitivity (90%) and specificity (65%).

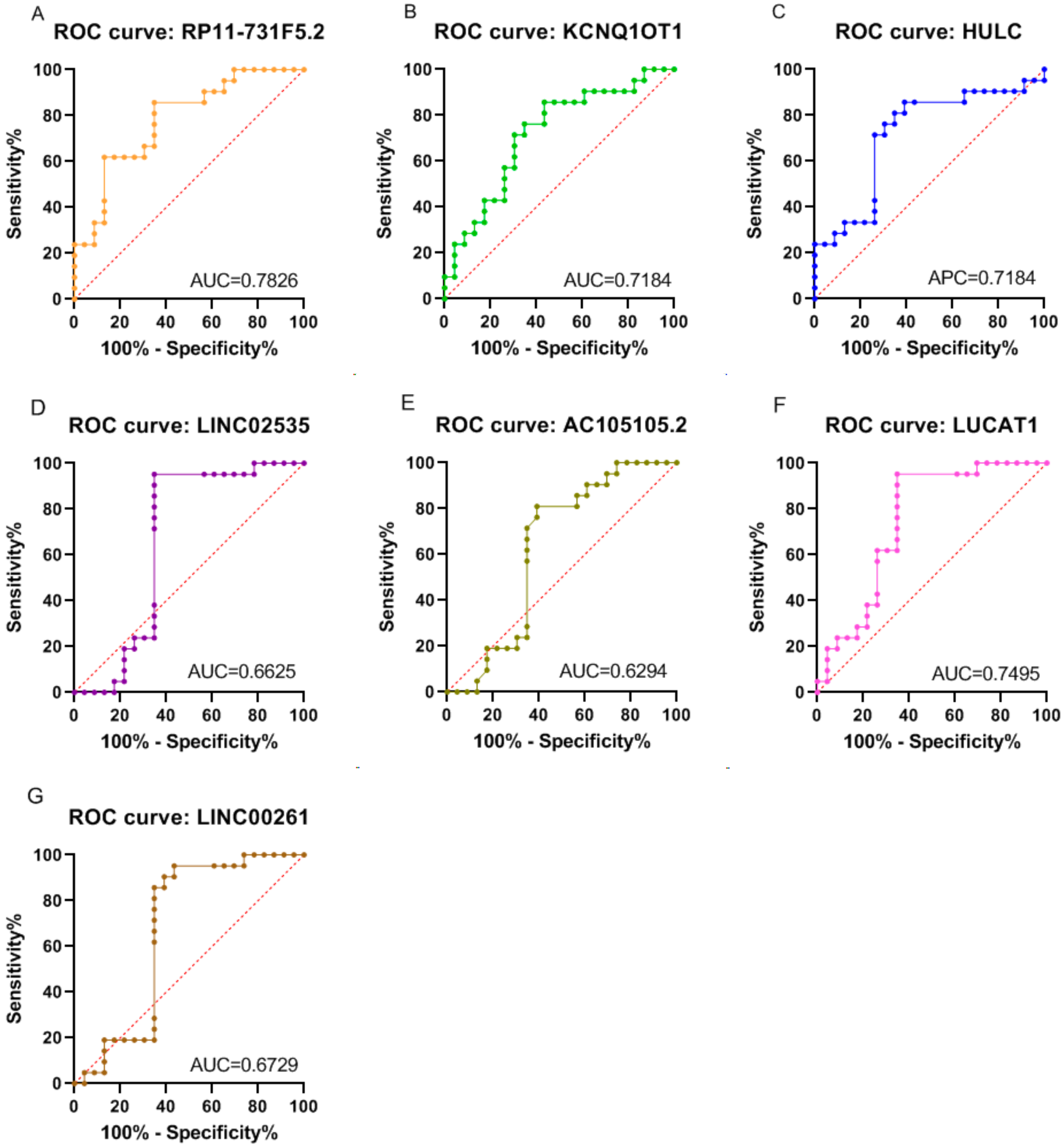

Figure S3B. ROC Curve Analysis for lncRNAs in Plasma. CHCneg *versus* DS groups; IC = 95%; AUC: Area Under Curve; The red dashed line represents the reference line (nullity). This analysis and graphs were performed in GraphPad Prism version 9.5.1. A) RP11-731F5.2 (AUC = 0.7826, p = 0.013), sensitivity (86%) and specificity (65%); B) KCNQ1OT1 (AUC = 0.7184, p = 0.0132), sensitivity (86%) and specificity (56%); C) HULC (AUC = 0.7184, p = 0.0132), sensitivity (86%) and specificity (61%); D) LINC02535 (AUC = 0.6625, p = 0.0651), sensitivity (90%) and specificity (57%); E) AC105105.2 (AUC = 0.6294, p = 0.1420), sensitivity (86%) and specificity (65%); F) LUCAT1 (AUC = 0.7457, p = 0.0046), sensitivity (95%) and specificity (65%); G) LINC00261 (AUC = 0.6729, p = 0.0498), sensitivity (95%) and specificity (65%).

Table S1. Performance of isolated and combined* lncRNAs as biomarkers of hepatocellular carcinoma risk in blood plasma of patients with chronic hepatitis C

Combinations	HCCneg *vs* HCCpos		
	AUC	SE (%)	SP (%)
**RP11-731F5.2**	**0.669**	**80**	**62**
RP11-731F5.2/KCNQ1OT1	0.605	95	38
RP11-731F5.2/HULC	0.726	90	62
RP11-731F5.2/LINC02535	0.621	95	38
RP11-731F5.2/AC105105.2	0.612	95	38
RP11-731F5.2/LUCAT1	0.662	85	57
RP11-731F5.2/LINC00261	0.624	70	67
RP11-731F5.2/AFP	0.617	85	38
**KCNQ1OT1**	**0.612**	**70**	**57**
KCNQ1OT1/HULC	0.779	100	62
KCNQ1OT1/LINC02535	0.610	65	62
KCNQ1OT1/AC105105.2	0.633	80	57
KCNQ1OT1/LUCAT1	0.590	70	57
KCNQ1OT1/LINC00261	0.617	62	85
KCNQ1OT1/AFP	0.624	85	43
**HULC**	**0.726**	**73**	**72**
HULC/LINC02535	0.733	90	62
HULC/AC105105.2	0.748	90	67
HULC/LUCAT1	0.688	75	62
HULC/LINC00261	0.731	85	62
HULC/AFP	0.705	75	71
**LINC02535**	**0.586**	**65**	**72**
LINC02535/AC105105.2	0.648	70	76
LINC02535/LUCAT1	0.560	80	47
LINC02535/LINC00261	0.674	80	76
LINC02535/AFP	0.562	25	95
**AC105105**	**0.669**	**75**	**76**
AC105105.2/LUCAT1	0.664	80	67
AC105105.2/LINC00261	0.686	80	76
AC105105.2/AFP	0.607	90	33
**LUCAT1**	**0.552**	**65**	**57**
LUCAT1/LINC00261	0.633	80	62
LUCAT1/AFP	0.555	35	86

**LINC00261**	**0.671**	**80**	**76**
LINC00261/AFP	0.581	95	24
**AFP**	**0.569**	**85**	**33**

*Only $p > 0.05$; SP: specificity; SE: sensitivity; AFP: Alpha-fetoprotein; AUC: Area Under Curve; HCCneg: patients with CHC who did not develop HCC within 5 years of follow-up; HCCpos: patients with CHC who developed HCC within 5 years of follow-up
